# Seeds of hope: Mucuna Pruriens offers promise for persons with Parkinson's disease in Africa

**DOI:** 10.1177/1877718X251394771

**Published:** 2025-11-11

**Authors:** Nicola Modugno, Natasha Fothergill-Misbah

**Affiliations:** 1 IRCCS Neuromed, Pozzilli, IS, Italy; 2 Population Health Sciences Institute, Newcastle University, Newcastle upon Tyne, UK

The new study on Mucuna pruriens from Cilia and colleagues, that was published in this issue of The Journal of Parkinson's Disease,^1^ represents a true milestone for the management of Parkinson's disease in low- and middle-income countries, where access to conventional levodopa-based medications is often extremely limited or prohibitive for patients.^2,3^ Mucuna pruriens is a naturally growing tropical leguminous plant, the beans of which contain levodopa. Beans are roasted, ground into powder and dissolved in water for consumption.

The multicenter, randomized, controlled trial conducted in Ghana demonstrates, for the first time with 12-month data, the non-inferiority of Mucuna pruriens powder compared to standard treatment with levodopa + dopa decarboxylase inhibitor, both in terms of motor and non-motor efficacy and safety. The study's objective is highly relevant from a public health perspective, as it addresses the problem of levodopa accessibility across Africa and offers a local and economically sustainable solution.

The results were consistent and robust across all key endpoints: Mucuna pruriens powder showed similar efficacy to levodopa/DDCI on motor symptoms, non-motor symptoms, and quality of life; the rate of adverse events was similar, most of which were mild. The paper comes with compelling video footage of patients before and after intake of Mucuna pruriens, showing at times impressive improvements (the videos can be found on the journal's website). The main strength of the study is the demonstration that a natural, economically sustainable, and locally cultivated solution can be successfully implemented under careful medical supervision, restoring quality of life and therapeutic dignity even to those millions of people living in areas where the “gold standard” pharmacological treatment is simply impossible to find or afford.

From a methodological perspective, the randomized controlled design and the length of follow-up gives robustness to the results: both arms showed similar improvements on the PDQ-39, UPDRS, and non-motor symptoms, with a tendency toward fewer severe adverse events, even without any real statistical differences. Additional notable features include the fact that the study protocol was sensitive to the African context, including flexibility in preparing and consuming Mucuna pruriens (dissolved powder or supernatant), and the compliance with clinical ethics.

There are, however, some limitations: the first concerns the relatively small number of patients recruited, necessitated by the COVID-19 pandemic and the logistical difficulty of finding truly drug-naïve subjects in a context where the diagnosis often occurs late—largely due to the widespread unavailability of neurology specialists, limited awareness about Parkinson's disease and stigma associated with symptoms. Furthermore, the open-label nature of the study may have introduced some bias, while the generalizability of the results outside of Ghana—although supported by similar data obtained in Bolivia and Thailand—will need to be verified with future, larger, multicenter cohorts. Another critical aspect is the greater number of gastrointestinal side effects with Mucuna, which were fortunately almost always mild and transient and manageable with technical precautions in preparation. For all these reasons, there is the need of future specific comparative pharmacokinetic studies between MP and levodopa/DDCI to better understand bioequivalence and individual variability. These issues are the main gaps that research should try to fill in the future.

Understanding the real effective role of Mucuna would be extremely important because the potential global impact of these data is enormous: if confirmed by larger studies with extended follow-ups, the ability to cultivate, process, and use Mucuna pruriens in a standardized manner and under clinical supervision would represent a turning point for countries with under-funded and/or less structured healthcare systems, reducing inequalities in access to treatment and freeing patients from the burden of unaffordability. In addition to improving public health, promoting local Mucuna production chains would have social and economic benefits for the communities involved. However, this should not detract from the need to continue advocating—at a grassroots and policy level—for improved access to levodopa/DDCI for all patients in Africa.

Mucuna pruriens in supplement form is already accessed by persons with Parkinson's seeking more ‘natural’ treatment in high-income countries. However, commercially available capsules vary in dosage and patients often self-medicate by adding Mucuna pruriens to prescribed levodopa/DDCI, potentially influencing clinicians’ optimization of therapy.^4^ As such, where levodopa/DDCI is available, accessible and affordable—such as in high-income countries—use of Mucuna would not be advised in place of prescribed treatments.

Taken together, this new article lays a solid scientific foundation for a concrete paradigm shift in healthcare policy, giving voice and hope to millions of patients currently forgotten in global statistics.
